# Economic NMPC for a Reversible Solid Oxide Cell

**DOI:** 10.1021/acs.iecr.5c04217

**Published:** 2026-01-02

**Authors:** Sakshi S. Naik, Yufei Zhao, Douglas Allan, Sangbum Lee, Lorenz T. Biegler

**Affiliations:** † 6612 Carnegie Mellon University, Pittsburgh, Pennsylvania 15213, United States; ‡ 311308 Purdue University, West Lafayette, Indiana 47907, United States; § 17213 National Energy Technology Laboratory, Pittsburgh, Pennsylvania 15236, United States; ∥ NETL Support Contractor, Pittsburgh, Pennsylvania 15236, United States

## Abstract

Reversible solid oxide fuel cells (rSOCs) offer the flexibility to operate in tandem with the electric grid by switching between fuel cell and electrolysis modes based on real-time electricity prices. However, their complex, tightly coupled dynamic behavior poses significant challenges in determining optimal operating strategies. In this work, we present an economic nonlinear model predictive control (E-NMPC) framework to optimize the operation of rSOCs. The proposed E-NMPC is applied to a detailed rSOC flowsheet model that includes a utility scale rSOC module as well as balance-of-plant equipment necessary for thermal management. Our results demonstrate that in fuel cell mode, the E-NMPC strategy reduces hydrogen consumption compared to conventional set-point tracking NMPC, while maintaining the same level of electricity output. Also, in electrolysis mode, the E-NMPC yields a marginal improvement in hydrogen production. In addition, we explore the integration of a battery with the rSOC system to enhance flexibility in meeting electricity production and consumption targets.

## Graphical abstract

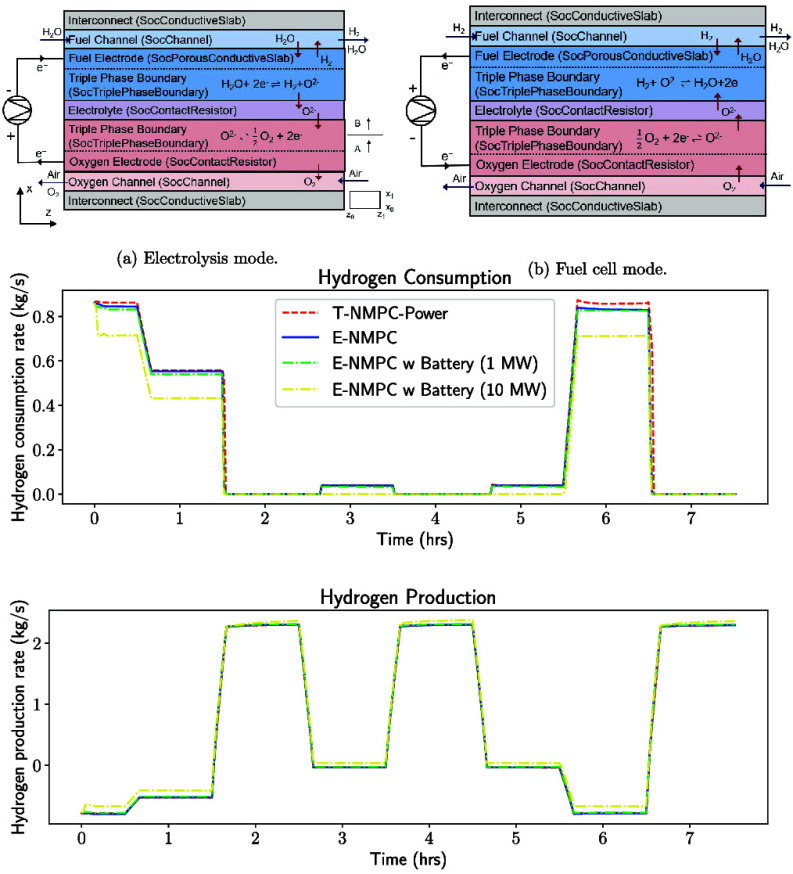

## 1 Introduction

The solid oxide fuel cell (SOFC) market has grown significantly in recent years as a result of the pressing need for energy diversification.^15,24^ SOFC converts chemical energy from a variety of fuel sources, such as hydrogen, to electrical energy. In reverse mode, a solid oxide fuel cell becomes a solid oxide electrolysis cell (SOEC), which acts as a producer of high-purity hydrogen by electrolysis of water.^25^ A reversible solid oxide cell (rSOC) can operate in both fuel cell mode and electrolysis mode. Reversible operation is desirable to meet the fluctuating market and prices of electricity. In the United States, locational marginal pricing (LMP) of electricity is determined by a variety of factors such as power generation, demand, and transmission system constraints.^4^ It is profitable to produce electricity when the LMP is high and to produce hydrogen for both future consumption and sale when the LMP is low. An rSOC can meet these fluctuating electricity demands by alternating between electricity production and consumption to support the grid.

The rSOC is a complex, dynamic system with strongly coupled electrochemical, thermal, and mechanical processes. In addition, an rSOC stack is connected to several other modules such as heat exchangers, a flash vessel, splitters, mixers, etc., that are used for thermal management and separation of hydrogen gas and water. Operating this complex system efficiently requires advanced modeling and optimization approaches. Therefore, mathematical models of the rSOC have been developed at different scales and complexities. Several researchers have developed steady-state models and have validated them against experimental data.^2,19,33^ Although these models provide insight into the physics of the system at steady state, they do not capture the system transients that are crucial to understanding cell operation. Jin and Xue^13^ investigated the transient behavior of rSOC by developing an isothermal 2D transient model to understand the internal mechanism of the cell during mode switching. Their study indicated that the trends of internal parameter distributions, including ionic potentials, flip when the operating mode is switched from SOFC mode to SOEC mode. More recently, Wang et al.^26^ simulated the performance of the rSOC stack under adiabatic conditions, providing new insights into the design and operation of the stack, indicating that higher operation pressure and lower current density are beneficial for increasing the reversible efficiency of the SOC stack.

Researchers have demonstrated the effectiveness of advanced control strategies for both SOEC and SOFC systems. For example, Cai et al.^3^ developed optimal control strategies for a 1D SOEC model coupled with an air compressor to optimize hydrogen production. Bhattacharyya and Rengaswamy^1^ proposed a high-fidelity dynamic model of an SOFC, which was incorporated into a nonlinear model predictive control (NMPC) framework to achieve step changes in load with acceptable overshoots in power and fuel utilization. Similarly, Spivey et al.^23^ designed a MIMO predictive controller for a tubular SOFC that demonstrated effective load-following capabilities. Robust control techniques have also been explored; Wu et al.^27^ applied such a strategy to an SOFC flowsheet under parametric uncertainties, while higher-order sliding mode control was shown to effectively regulate stack temperature under noisy load conditions.

The application of optimal control strategies to rSOCs remains relatively underexplored. This gap is significant given the operational complexities involved in transitioning between SOEC and SOFC modes. Addressing this challenge, Li et al.^17^ recently implemented a nonlinear model predictive control framework for an rSOC flowsheet to track set-points for hydrogen production and consumption; they showed the benefits of NMPC over multiloop PID controllers with respect to enforcing process constraints and providing smoother transitions at switching points. In this work, an upper-level steady-state optimization model was used to generate the optimal hydrogen production and consumption set-points based on the LMP of electricity and assuming a fixed price of hydrogen. However, set-point tracking NMPC can be suboptimal for a dynamic system. Alternatively, economic NMPC directly optimizes an economic cost function to find the optimal control actions.^21^ Economic NMPC has been shown to perform better than set-point tracking NMPC for a variety of applications.^5,8,14,22,32^

In this paper, we extend the work of Li et al.^17^ to utilize an economic NMPC to find cost-optimal control actions for operating the rSOC flowsheet. The economic NMPC directly minimizes hydrogen consumption in the SOFC mode and maximizes hydrogen production in the SOEC mode while meeting electricity demand.^7^ We demonstrate the economic NMPC framework on an rSOC flowsheet using a realistic LMP for electricity and compare it against set-point tracking NMPC to highlight key operational differences.

## 2 Process Modeling

An rSOC can operate in both electrolysis and fuel cell modes. In electrolysis mode (Figure 1a), steam is fed into the fuel channel and transported to the triple phase boundary (TPB), where the electrode (electronic conductor), electrolyte (ionic conductor) and gas intersect.^1^ At the fuel side of the TPB, electrons are consumed to reduce steam; thus, it acts as the cathode. Oxygen ions are generated and subsequently transported through the electrolyte and oxidized at the oxygen side of the TPB to release electrons; thus, it acts as the anode. Product O_2_ is blown out of the unit via the sweep stream.

**Figure 1:**
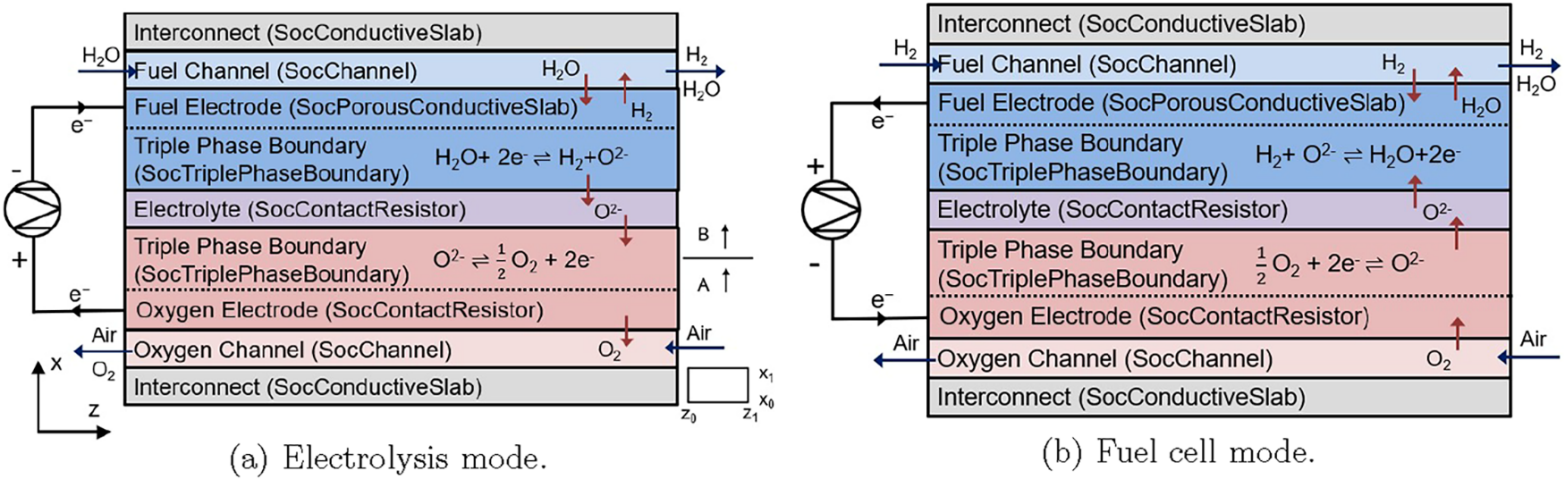
Diagram for reversible solid oxide cell (rSOC) model.

In fuel cell mode (Figure 1b), H_2_ is fed to the fuel channel. The roles of the electrode are interchanged; the cathode in electrolysis mode acts as the anode in fuel cell mode, where H_2_ reacts with transported oxygen ions to form steam and release electrons. In contrast, the electrode acting as the anode in electrolysis becomes the cathode, where oxygen ions are produced from O_2_. Referring to the anode and cathode in an rSOC is ambiguous because the role the electrode plays flips between fuel cell and electrolysis modes. Thus, the fuel electrode refers to where H_2_ is produced or consumed, while the oxygen electrode is where oxygen is produced or consumed.^17^

### 2.1 SOC Model

A detailed nonlinear dynamic model of the rSOC is fully developed and described in.^6,17^ A single rSOC in Figure 1 is composed of five main submodels that are modeled as unit models in IDAES.^16^

1. Channel (fuel and oxygen channels)
2. Conductive slab (Interconnect)
3. Porous conductive slab (fuel electrode)
4. Contact resistor (electrolyte and oxygen electrode)
5. Triple phase boundary

The model consists of partial differential equations (PDEs) with boundary conditions. Table 1 summarizes the model equations developed in ref 17, and all variables are defined in the nomenclature. The gas is assumed to be an ideal gas, modeled using the ideal gas law. The channel material and energy balances are first-order DAEs. The conductive slab is discretized in both the *x* and *z* directions, with 1 finite element in the *x*-direction resulting in a quasi-1D PDE system. As it is a solid phase, it does not involve a material balance. The energy balance accounts for Joule heating and thermal conduction. In the porous conductive slab, ε is the void fraction of the electrode. The energy transport resembles that of the conductive slab, with an additional term resulting from material flux in the void space. In contrast, mass transport occurs only in the gas phase via diffusion. The contact resistor has negligible thickness, and, therefore, is not extended in the *x* direction. Because the incoming and outgoing fluxes are not the same, they are defined separately at the boundaries. The TPB model is similar to the contact resistor model except that it has material and energy balance equations due to the reactions that occur in the TPB.

**Table 1:** rSOC Models

model	mechanism	equation
gas properties	ideal gas law	$\rho_{\mathrm{mol}}=\frac{P}{R_{u}T}$
	species balance	$C_{i}=y_{i}\rho_{\mathrm{mol}},\sum_{j}y_{j}=1$
channel model	material balance	$0=-\frac{\partial }{\partial z}\left(vC_{i}\right)+\frac{1}{L_{x}}\left(J_{i,x_{0}}-J_{i,x_{1}}\right)$
	energy balance	$0=-\sum_{i}\frac{\partial \left(J_{i,z}H_{i}\right)}{\partial z}+\frac{1}{L_{x}}\sum_{i}\left(J_{i,x_{0}}H_{i,x_{0}}-J_{i,x_{1}}H_{i,x_{1}}\right)+\frac{h}{L_{x}}\left(T_{x_{0}}-2T+T_{x_{1}}\right)$
	boundary conditions	$\mathrm{fuel}:\;J_{i}{|}_{z_{0}}L_{x}L_{y}N_{\mathrm{cell}}=F_{i,\mathrm{in}};q{|}_{z_{0}}L_{x}L_{y}N_{\mathrm{cell}}=\sum_{i}F_{i,\mathrm{in}}H_{i,z_{0}}$
		$$\mathrm{oxygen}\;J_{i}{|}_{z_{1}}L_{x}L_{y}N_{\mathrm{cell}}=-F_{i,\mathrm{in}};q{|}_{z_{1}}L_{x}L_{y}N_{\mathrm{cell}}=-\sum_{i}F_{i,\mathrm{in}}H_{i,z_{1}}$$
conductive slab	energy balance	$\frac{\partial \rho_{e,s}}{\partial t}=\rho j^{2}+k\left(\frac{\partial^{2}T}{\partial x^{2}}+\frac{\partial^{2}T}{\partial z^{2}}\right)$
	boundary conditions	${\frac{\partial T}{\partial z}|}_{z_{0}}=0;{\frac{\partial T}{\partial z}|}_{z_{1}}=0{T|}_{x_{0}}=T_{\mathrm{in}};\;{T|}_{x_{1}}=T_{\mathrm{out}}$
porous conductive slab	material balance	$0=-\frac{\partial J_{i,x}}{\partial x}-\frac{\partial J_{i,z}}{\partial z}$
	energy balance	$\left(1-\varepsilon \right)\frac{\partial \rho_{e,s}}{\partial t}=\frac{\rho }{1-\varepsilon }j^{2}+\left(1-\varepsilon \right)k\left(\frac{\partial^{2}T}{\partial x^{2}}+\frac{\partial^{2}T}{\partial z^{2}}\right)-\frac{\partial }{\partial x}\left(\sum_{i}J_{i,x}H_{i}\right)-\frac{\partial }{\partial z}\left(\sum_{i}J_{i,z}H_{i}\right)$
	boundary conditions	${C_{i}|}_{x_{0}}=C_{i,\mathrm{in}};{C_{i}|}_{x_{1}}=C_{i,\mathrm{out}};{\frac{\partial C_{i}}{\partial z}|}_{z_{0}}=0;{\frac{\partial C_{i}}{\partial z}|}_{z_{1}}=0$
		$${T|}_{x_{0}}=T_{\mathrm{in}};{T|}_{x_{1}}=T_{\mathrm{out}};{\frac{\partial T}{\partial z}|}_{z_{0}}=0;{\frac{\partial T}{\partial z}|}_{z_{1}}=0$$
contact resistor	heat flux model	$q_{B}=q_{A}+\rho lj^{2}$
triple phase boundary	material balance (fuel)	$J_{i,A}=0,J_{i,B}=\nu_{i,\mathrm{ftpb}}r_{\mathrm{ftpb}}$
	material balance (O_2_)	$J_{i,A}=\nu_{i,\mathrm{otpb}}r_{\mathrm{otpb}},J_{i,B}=0$
	energy balance	$q_{B}=q_{A}+j\eta_{\mathrm{act},k}-r_{k}T\Delta S_{\mathrm{rxn},k}$

### 2.2 Flowsheet Model

The rSOC model developed in the previous section is part of a larger flowsheet shown in Figure 2. The cell unit is part of a module composed of *N*_cell_ cells. The single cell model, which represents a cell in the interior of the module, is used to approximate the behavior of all cells in the module. In Figure 2, the operation of the flowsheet in fuel cell mode is shown in blue and in electrolysis mode is shown in red. The product streams from the rSOC can be recycled back to the stack or vented out of the system. In fuel cell mode, the makeup fuel gas stream consists of 96.9% H_2_, 3% H_2_O and 0.1% inert gases. In addition, the flash unit is used to knock out water from the hydrogen-rich fuel gas from the cell stack to mix the hydrogen with the makeup stream. In electrolysis mode, the makeup stream consists of 99.9% H_2_O and 0.1% inert gases. The hydrogen-rich gas after the flash is vented out of the system. In both modes, air is used in the sweep stream and recycled using the sweep splitter or vented out of the process.

**Figure 2:**
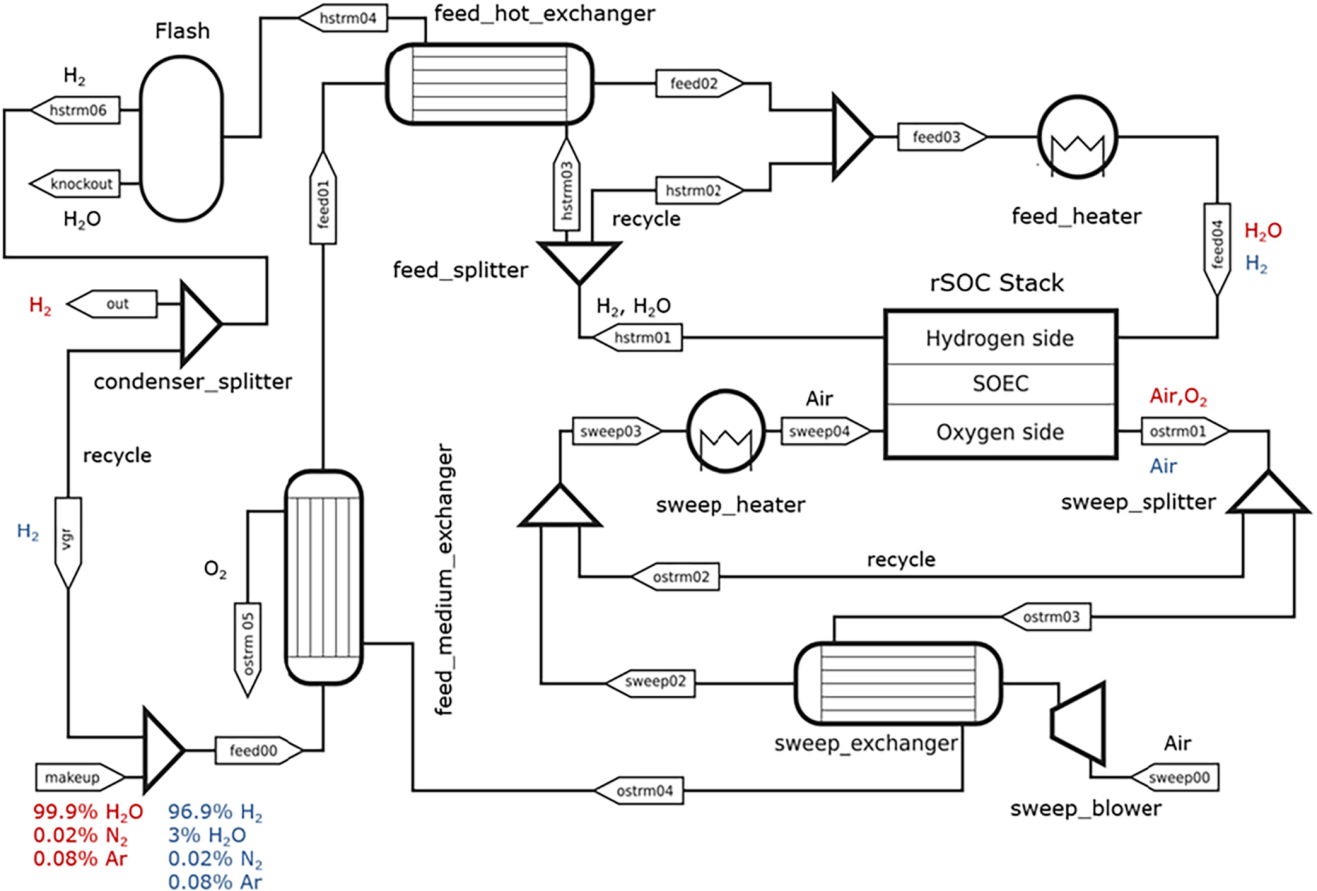
Process diagram of rSOC system, where the red labels correspond to operation in electrolysis mode, while the blue labels correspond to operation in fuel cell mode.

The flowsheet also contains three heat exchangers for heat integration. The hot product streams from the rSOC stack are used to preheat the feed and sweep streams. Two trim heaters (feed_heater and sweep_heater in Figure 2) provide additional heat to the inlet streams of the stack. The net energy consumption of the flowsheet is the combined electricity consumed by the rSOC stack, the trim heaters, and the sweep blower. Heat exchangers and trim heaters use a 1D model, while the remaining supporting equipment units use the 0D models.^17^ The system uses cross-flow heat exchangers with boundary conditions specified at inlets of the shell and tube side.

#### 2.2.1 Discretization Analysis of Heat Exchangers

The rSOC flowsheet model must remain numerically stable under varying parameter values. However, maintaining convergence becomes particularly challenging when the system switches between fuel cell and electrolysis modes.

While troubleshooting convergence issues for E-NMPC, it was discovered that the discretization used for the cross-flow heat exchangers was numerically unstable. Naïve use of backward finite differences for both the shell side (which flows from *z* = 0 to *z* = 1) and the tube side (which flows from *z* = 1 to *z* = 0) caused nonphysical oscillations in temperature. Replacement with an upwind difference scheme, using a backward difference for the shell side and a forward difference for the tube side, resulted in an error in the energy balance proportional to the discretization step length.[Fn fn1] Instead, a spatially symmetric Lagrange-Legendre collocation scheme was found to be numerically stable and conserve energy.

#### 2.2.2 Variable Transformation

Variable transformations can improve the numerical stability of optimization problems, preventing issues such as division by zero or large condition numbers of the constraint Jacobian or model Hessian. Specifically, in the rSOC flowsheet, several variable transformations were applied to make the model robust. One of the transformations was based on the definition of the recycle ratio from the split fraction. For a split fraction *s* to a recycle stream, the recycle ratio *r* is defined as follows

$$r=\frac{s}{1-s}$$

During rSOC mode switching, the split fraction *s* can approach 1, which makes the recycle ratio undefined and leads to singularities in the model and convergence problems. Therefore, the definition of the recycle ratio was reformulated as follows

$$s=\frac{r}{r+1}$$

This reformulated definition of the recycle ratio and the split fraction is well-defined for values of *s* → 1 and no longer leads to singularities in the model.

## 3 NMPC Formulation

In this section, several NMPC formulations are developed for the rSOC model to handle real-world electricity prices. The locational marginal price of electricity fluctuates frequently in a day. Advanced control methods enable optimal operation and transition between different operating modes of the rSOC depending on the LMP. When the LMP of electricity is high, it is desirable to operate the SOC in fuel cell mode to consume hydrogen and produce electricity. When the LMP of electricity is low, the SOC is operated in electrolysis mode, where hydrogen is produced by consuming electricity. At certain intermediate LMP values, neither the fuel cell nor electrolysis modes are profitable. However, a full shutdown of the rSOC takes several hours and puts the cell under great thermal stress. To avoid such a shutdown, a third “hot idle” state is used, in which a minimum amount of steam and hydrogen is fed into the system to keep it warm and avoid thermal cycling. In this section, the set-point tracking and economic NMPC formulations are described in detail.

### 3.1 Tracking NMPC (T-NMPC-Hydrogen)

As a baseline, the set-point tracking NMPC from Li et al.^17^ is presented. The steady-state SOC model is optimized based on the LMP data to obtain the optimal steady-state set-points used in the set-point tracking NMPC. The objective function switches on the basis of whether the SOC is in fuel cell mode or electrolysis mode. In fuel cell mode, the objective function tracks the H_2_ consumption from the steady-state set-point, and in electrolysis mode, it tracks the H_2_ production. The objective function for the control horizon of length *N* can be written as follows:

1 $$l^{\mathrm{tr}}=\sum_{k=0}^{N}\left(\gamma^{c}\left(k\right){\left[H_{2}^{c}\left(k\right)-H_{2}^{c,\mathrm{sp}}\left(k\right)\right]}^{2}+\gamma^{p}\left(k\right){\left[H_{2}^{p}\left(k\right)-H_{2}^{p,\mathrm{sp}}\left(k\right)\right]}^{2}+\lambda \sum_{w\in \mathrm{MV}\cup \mathrm{CV}}{\left[w\left(k\right)-w{\left(k\right)}^{\mathrm{sp}}\right]}^{2}\right)$$

Hydrogen consumed in time period *k* in fuel cell mode is given by H_2_^*c*^, and the corresponding hydrogen consumption set-point derived from the steady-state optimization is given by H_2_^*c*,sp^. The coefficient γ^*c*^ is a parameter that takes the value of 1 when the SOC is in fuel cell mode, and otherwise it is 0. Similarly, H_2_^*p*^ is the hydrogen produced in the electrolysis mode and H_2_^*p*,sp^ is the corresponding hydrogen production set-point.[Fn fn2] The parameter γ^*p*^ takes the value of 1 in electrolysis mode and 0 otherwise. λ is a penalty parameter for deviation of manipulated and controlled variables from their set-points. The penalty term is necessary to fully define the system and make the controller stable.

### 3.2 Tracking NMPC (T-NMPC-Power)

Electricity markets are operated in a day-ahead fashion, and the amount of electricity that is committed to the market must be utilized.^7^ The set-point tracking controller from Li et al.,^17^ which tracks hydrogen targets, might not necessarily meet the electricity targets. Therefore, in this section, the set-point tracking objective is modified to track electricity targets along with hydrogen tracking. The objective function is given by

2 $$l^{\mathrm{tr}}=\sum_{k=0}^{N}\left(\gamma^{c}\left(k\right){\left[H_{2}^{c}\left(k\right)-H_{2}^{c,\mathrm{sp}}\left(k\right)\right]}^{2}+\gamma^{p}\left(k\right){\left[H_{2}^{p}\left(k\right)-H_{2}^{p,\mathrm{sp}}\left(k\right)\right]}^{2}+\beta \left|\mathrm{TE}\left(k\right)-\mathrm{TE}{\left(k\right)}^{\mathrm{sp}}\right|+\sum_{w\in \mathrm{MV}\cup \mathrm{CV}}{\left[w\left(k\right)-w{\left(k\right)}^{\mathrm{sp}}\right]}^{2}\right)$$

Here, TE is the total electricity consumed or produced in the SOC, and TE^sp^ is the set-point obtained from steady-state optimization. The constraint on the utilization of the bid electricity is enforced using an $l_{1}$ penalty term with weight β in the objective function.

### 3.3 Economic NMPC (E-NMPC)

During transitions between set-points, a reference trajectory for tracking NMPC is created by interpolating between steady-state values. However, this trajectory is not necessarily optimal. Additionally, for the SOC system, the set-point for the transition between two steady states is determined by interpolation, which is not necessarily the most economic path. Economically, it is desired to minimize H_2_ consumption in the fuel cell mode and maximize H_2_ production in the electrolysis mode. Therefore, the economic NMPC directly minimizes H_2_ consumption in the fuel cell mode and maximizes H_2_ production in the electrolysis mode while meeting the electricity production and consumption targets. The economic NMPC objective function for a controller with horizon T is given by

3 $$l^{\mathrm{ec}}=\sum_{k=0}^{N}\left(\gamma^{c}\left(k\right)H_{2}^{c}\left(k\right)-\gamma^{p}\left(k\right)H_{2}^{p}\left(k\right)+\beta |\mathrm{TE}\left(k\right)-TE^{\mathrm{sp}}\left(k\right)|+\lambda \sum_{w\in \mathrm{MV}\cup \mathrm{CV}}{\left[w\left(k\right)-w{\left(k\right)}^{\mathrm{sp}}\right]}^{2}\right)$$

The penalty term for meeting the reference points on the MVs and CVs can be adjusted using the tuning parameter λ. A lower value of λ leads to a higher economic benefit; however, the stability of the controller may be compromised.^8–12,22^

### 3.4 E-NMPC for SOC with a Simplified Battery Model

The constraint on exactly producing or consuming electricity that is committed to the market can be overly restrictive, especially when an alternative source of energy is available to produce or consume some of the electricity. In this section, we formulate the objective function assuming that an alternative electricity source, such as a battery, is available to handle some load of the SOC. In practice, an SOC coupled with a battery creates a hybrid energy system in which the battery is used as a buffer to maintain stable operation of the SOC under fluctuating loads.^29^ Note that in this work, the battery is not rigorously modeled but simplified as an alternative source/sink of electricity with a large capacity and negligible capital and operating cost. The electricity commitment is met using both the electricity generated by the SOC and the electricity provided by the battery. The objective function is defined as follows:

4 $$l^{\mathrm{ec}}=\sum_{k=0}^{N}\left(\gamma^{c}\left(k\right)H_{2}^{c}\left(k\right)-\gamma^{p}\left(k\right)H_{2}^{p}\left(k\right)+\beta \left|\mathrm{TE}\left(k\right)+\mathrm{BF}\left(k\right)-TE^{\mathrm{sp}}\left(k\right)\right|+\lambda \sum_{w\in \mathrm{MV}\cup \mathrm{CV}}{\left[w\left(k\right)-w{\left(k\right)}^{\mathrm{sp}}\right]}^{2}\right)$$

The battery flow BF takes both positive and negative values. The battery charges when the battery flow is positive and discharges when the battery flow is negative. In this formulation, there is no implicit incentive to charge the battery unless the controller determines that it is an optimal decision due to the predicted electricity demand within the control horizon. Therefore, it is assumed that the battery capacity is large enough so that it does not completely discharge during the SOC simulation. There is no guarantee that the electricity used to charge the battery is equal to the electricity discharged from the battery in the long run.

## 4 Results and Discussion

The results are divided into four sections. Section 4.1 compares the baseline set-point tracking NMPC formulation (T-NMPC-Hydrogen) against the set point tracking NMPC with $l_{1}$ penalty on power (T-NMPC-Power). Section 4.2 investigates the impact of the penalty parameter on set-point tracking terms on the MVs and CVs in the economic NMPC (E-NMPC) formulation. Section 4.3 compares the performance of set-point tracking NMPC against Economic NMPC and two E-NMPC battery configurations operating at different battery flow rates. Finally, Section 4.4 investigates the characteristic variables that lead to an improvement in the performance of E-NMPC compared to set-point tracking NMPC.

For all simulations, the time discretization was set to 75 s, and the controller horizon was 375 s, i.e., 5 time steps in the future. Longer horizons were also considered by trial and error in the construction and evaluation of the optimization model, but they did not lead to noticeable improvements in performance. Details on the problem sizes and solution times for each NMPC-based controller are given in Table 5.

Hourly LMP data from the NREL database (https://data.nrel.gov/submissions/181) was used for both steady-state optimization and E-NMPC. A section of LMP data in January in the $150 CAISO case was chosen so that frequent switching between fuel cell mode and electrolysis mode would occur.^9^ The control framework was implemented in Pyomo^10^ using the Pyomo MPC framework.^20^ Each instance of the controller model and the plant simulation was solved using the open source nonlinear solver IPOPT.^28^

To switch hydrogen consumption and production between different modes, it is necessary to determine when the out stream is rerouted from the hydrogen compression and drying train to a purge and vice versa. Here, the switch is chosen to occur when the “hydrogen production” set-point becomes negative—that is to say when it is forecast that more hydrogen enters the system through the makeup stream than leaves through the out stream. Because the set-points during the ramp are determined by interpolating between steady-state set-points, the power set-point and the hydrogen production set-point may indicate that, despite the fact that the system is actually operating in electrolysis cell mode, the out stream is purged. Ideally, either E-NMPC or some sort of dynamic optimization would choose the point when the out stream’s destination is switched, but the inclusion of this discrete decision is beyond the scope of this work.

### 4.1 Set-Point Tracking NMPC

The baseline T-NMPC-Hydrogen approach was compared with T-NMPC-Power, with the results summarized in Table 2. T-NMPC-Hydrogen strategy fails to meet the electricity committed in the market because T-NMPC-Hydrogen minimizes the distance from the hydrogen set-points but does not account for the electricity commitment. In contrast, T-NMPC-Power is able to meet the electricity commitment due to the $l_{1}$ penalty for meeting the set-point of electricity in the objective function (eq 2).

**Table 2:** SOC Performance When T-NMPC-Hydrogen and T-NMPC-Power Controllers are Used

	fuel cell mode		electrolysis mode	
	hydrogen consumed (kg)	electricity produced (MWh)	hydrogen produced (kg)	electricity consumed (MWh)
T-NMPC-hydrogen	6957.40	139.65	23,967.84	851.96
T-NMPC-power	6976.29	138.51	24,205.14	861.82
electricity commitment	-	138.51	-	861.82
hydrogen set-point	6974	-	24,057.09	-

The T-NMPC-Power consumed 0.3% more hydrogen compared to the T-NMPC-hydrogen to meet the electricity targets exactly. Similarly, in electrolysis mode, T-NMPC-Power consumed all the electricity that was bid and produced 1% more hydrogen compared to T-NMPC-Hydrogen. Overall, T-NMPC-Power operates the system in a manner that supports grid stability.

### 4.2 E-NMPC Parameter Tuning

The penalty parameter λ on the MVs and CVs is tuned to maximize the economic performance of the E-NMPC. Other parameters in the objective function, such as γ^*c*^, γ^*p*^ and β, are set to 1. The value of λ is gradually decreased, and the results are summarized in Table 3. The cumulative electricity production set-point was 138.51 MWh in the fuel cell mode, and the electricity consumption set-point was 861.82 MWh in the electrolysis mode. E-NMPC with λ = 1, 0.1, and 0.01 meets the electricity set-points, with different levels of hydrogen consumed and produced. As the value of λ decreases, the economic performance of the E-NMPC is improved. E-NMPC with λ = 1 and 0.1 consumed more hydrogen but produced less electricity than the case with λ = 0.01 due to a slightly lower conversion of hydrogen in the SOC stack—the surplus hydrogen left the system via the purge stream. Decreasing λ below 0.01 leads to stability issues in the controller.

**Table 3:** E-NMPC Performance with λ = 1, 0.1 and 0.01

	fuel cell mode		electrolysis mode	
	hydrogen consumed (kg)	electricity produced (MWh)	hydrogen produced (kg)	electricity consumed (MWh)
λ = 1	6876.14	137.39	24,227.01	861.81
λ = 0.1	6829.02	138.45	24,227.24	861.79
λ = 0.01	6696.85	138.51	24,286.13	861.82

E-NMPC with different values of λ is compared with T-NMPC-Power in Figure 3. E-NMPC with a higher value of λ performs close to T-NMPC-Power since the weight on the tracking terms is higher. However, the E-NMPC with smaller λ values can save hydrogen in the fuel cell mode and produce slightly more hydrogen in electrolysis. E-NMPC with λ = 0.01 is used in the next section for comparison against the set-point tracking NMPC cases since it is stable and provides maximum economic benefit.

**Figure 3:**
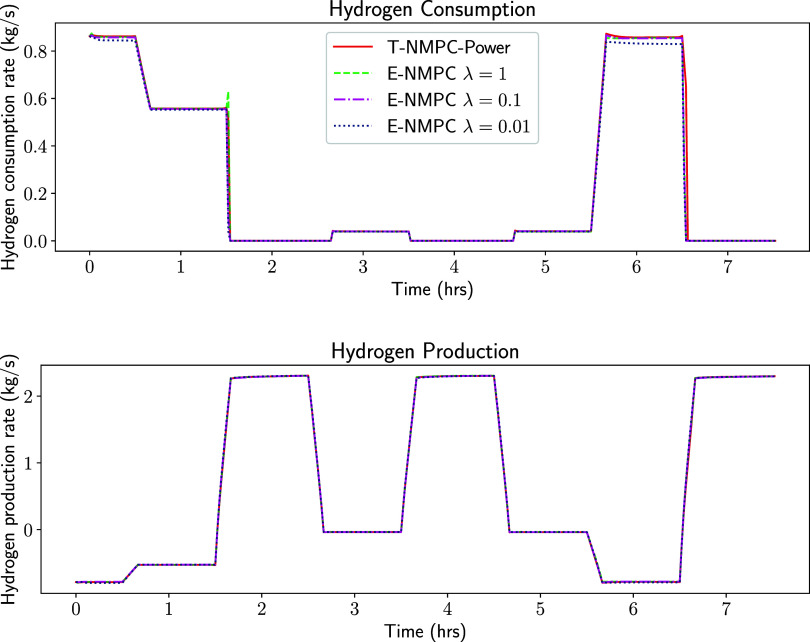
Hydrogen consumption, hydrogen production for economic NMPC with λ = 1, 0.1 and 0.01.

### 4.3 Tracking vs Economic NMPC

In this section, the set point tracking NMPC results are compared against the results from the economic NMPC and the economic NMPC with battery cases. Two experiments are conducted for the economic NMPC with battery:

1. E-NMPC with a simplified battery model with battery flow bounded between ±1 (MW)
2. E-NMPC with a simplified battery model with battery flow bounded between ±10 (MW)

Table 4 shows the net hydrogen consumption and electricity production in the fuel cell mode. In this case study, the total electricity production target was 138.51 MWh. The T-NMPC-Power case and E-NMPC case both meet the electricity production target. However, ≈4% less hydrogen was consumed in the E-NMPC case than in the T-NMPC-Power case. In the E-NMPC with 1 MW battery case, the hydrogen consumption was decreased by an additional 1.8% by satisfying some of the demand for electricity by using the battery. In the E-NMPC with 10 MW battery case, ≈26% less hydrogen was consumed compared to tracking NMPC; however, this is because a significant portion of the energy demand was satisfied by draining the battery instead of the SOC. Figure 4 compares the hydrogen consumption rate for all controllers. T-NMPC-Power consumed more hydrogen compared to that consumed in the E-NMPC cases, especially during the transition between fuel cell and electrolysis modes. E-NMPC was able to operate the cell at a slightly lower hydrogen consumption rate compared to tracking between 5.5–6.5 h.

**Figure 4:**
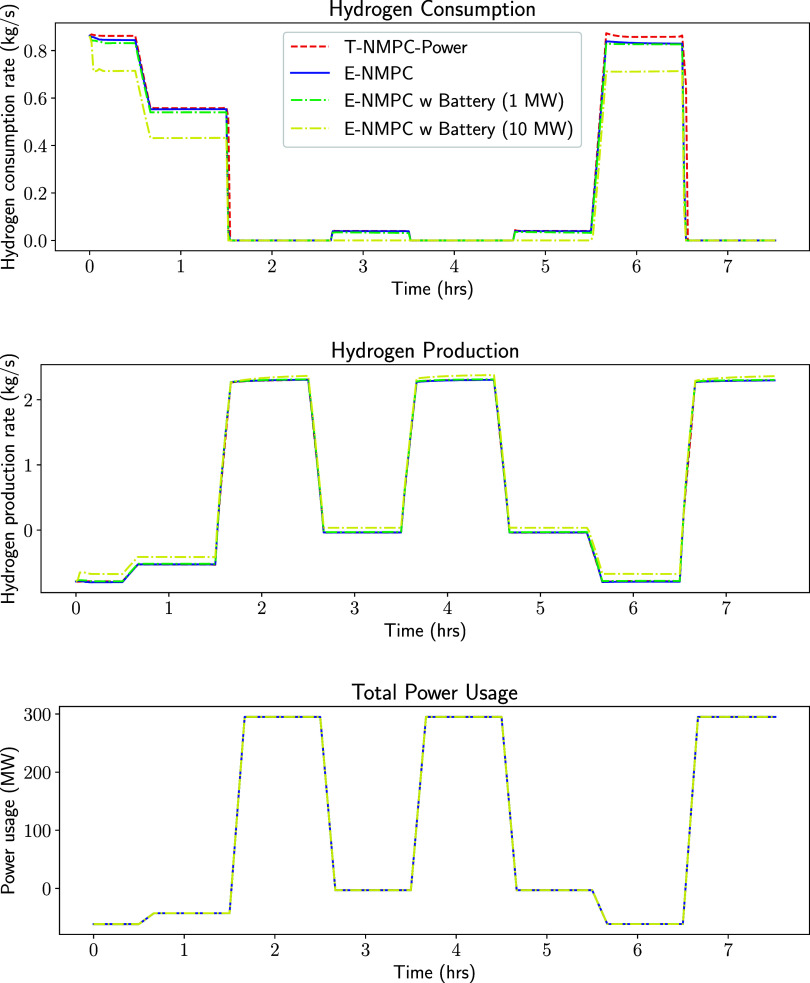
Hydrogen consumption, hydrogen production and total power usage economic NMPC with different penalty coefficient on variable tracking terms are used to control the SOC.

**Table 4:** Performance Comparison between T-NMPC-Power, E-NMPC and E-NMPC for SOC Coupled with a Battery

	fuel cell mode		electrolysis mode	
	hydrogen consumed (kg)	electricity produced (MWh)	hydrogen produced (kg)	electricity consumed (MWh)
T-NMPC-power	6976.28	138.51	24,205.14	861.82
E-NMPC	6696.85	138.51	24,286.12	861.82
E-NMPC w/battery (1 MW)	6570.57	138.51	24,366.88	861.82
E-NMPC w/battery (10 MW)	5345.62	138.51	25,110.52	861.85

In electrolysis mode, the economic NMPC produced slightly (≈0.3%) more hydrogen compared to that produced in the tracking NMPC case. Figure 4 shows that all controllers have similar hydrogen production rates. In the E-NMPC with the 10 MW battery case, hydrogen production was increased by ≈3.7% compared to the tracking NMPC. Figure 4 shows that the total power usage matches the set-point for all controllers, except T-NMPC-Hydrogen, which has lower power production while transitioning from the fuel cell mode to the electrolysis mode.

Finally, the NLP problem sizes and average computational performance for all NMPC-based controllers are given in Table 5 for all NMPC problem formulations.

**Table 5:** NLP Problem Size and Average Solve Time Per Controller Prediction Horizon (11th Gen Intel­(R) Core­(TM) i7-1165G7@2.80 GHz (2.80 GHz) with 16 GB RAM)

controller	variables	constraints	solve time (s)
T-NMPC	26,985	26,940	36.7
T-NMPC-power	26,997	26,946	37.7
E-NMPC	26,997	26,946	48.7
E-NMPC w/battery (1 MW)	27,008	26,951	50

### 4.4 Characteristic Variables

In this section, we analyze some key operational differences of the SOC between the T-NMPC-Power case and the E-NMPC with λ = 0.01 and no battery case. In fuel cell mode, the total electricity produced by the SOC stack must meet the committed grid electricity requirements, and, additionally, it must also generate extra electricity to operate the balance-of-plant equipment like the feed heater, sweep heater, and the sweep blower. In electrolysis mode, the electricity supplied by the grid is utilized in both the SOC stack for water electrolysis and the balance-of-plant equipment. Figure 5 shows that less electricity was consumed in the feed and sweep heaters in the E-NMPC cases compared to T-NMPC-Power. The electricity consumed in the sweep blower remained the same for both control approaches. As shown in Table 6, T-NMPC-Power consumed 1240 kWh of electricity to operate the feed heater and 2359 kWh to operate the sweep heater, while the E-NMPC consumed only 35 kWh of electricity to operate the feed heater and 11 kWh of electricity to operate the sweep heater. Therefore, the E-NMPC can reallocate the total available electricity such that more energy is allocated to the electrical work done in the SOC module. This reallocation results in reduced power generation requirements during fuel cell mode and increased electricity utilization by the SOC stack during electrolysis mode.

**Figure 5:**
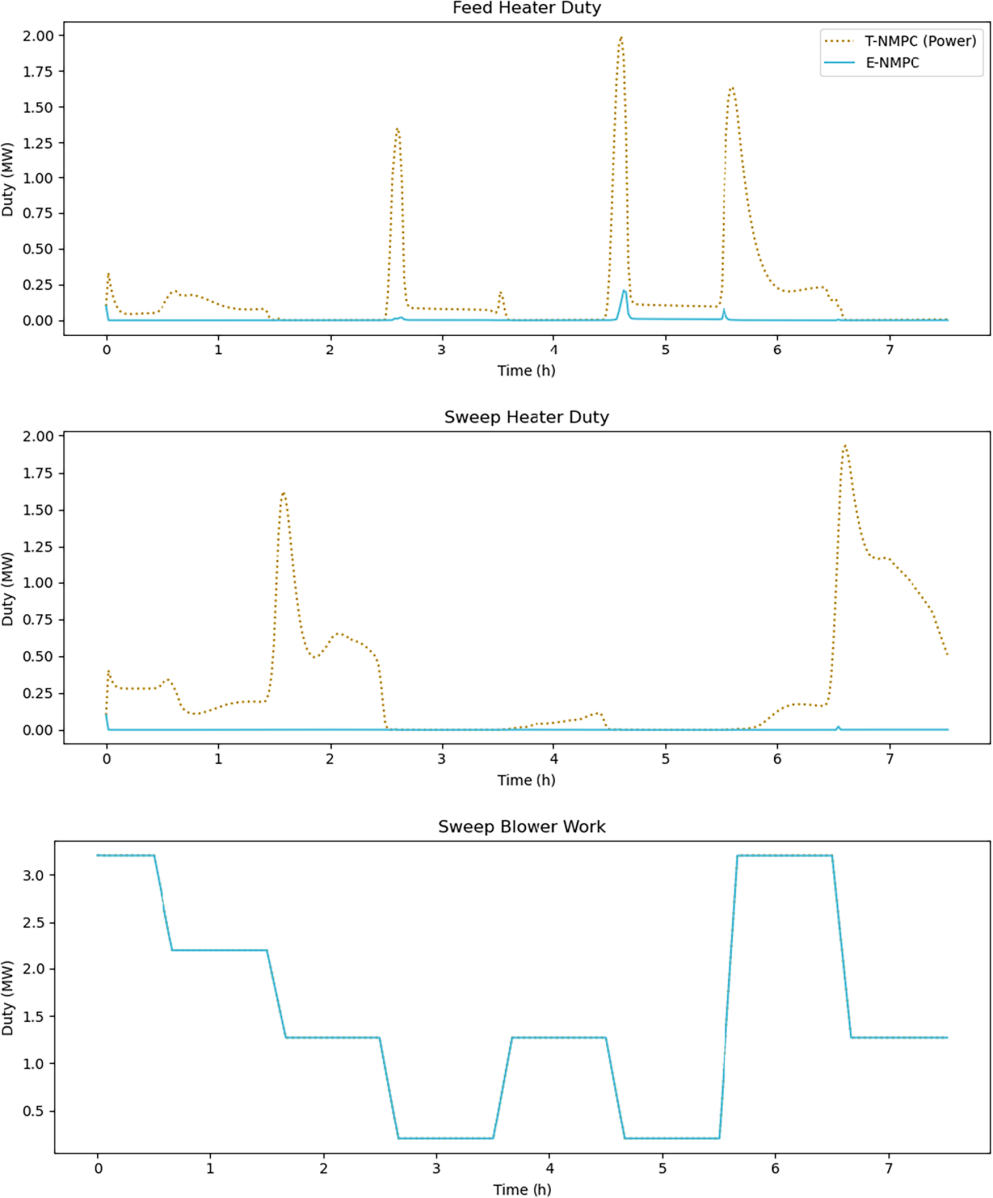
Electric heat duties for the feed and sweep heaters and sweep blower work for T-NMPC-Power and E-NMPC.

**Table 6:** Electricity Allocation in the SOC Flowsheet among the SOC Stack, Feed Heater, Sweep Heater, and Sweep Blower for the T-NMPC-Power and E-NMPC Control Strategies

	fuel cell mode					electrolysis mode				
unit (MWh)	electrical work	feed	sweep	blower	total	electrical work	feed	sweep	blower	total
T-NMPC-power	–147.217	0.846	0.499	7.365	–138.507	855.491	0.394	1.860	4.078	861.823
E-NMPC	–145.894	0.018	0.005	7.360	–138.511	857.723	0.017	0.006	4.077	861.823

The electrical work generated or consumed by the SOC is equal to the product of the cell potential and current. As shown in Figure 6, the SOC operated with the E-NMPC exhibits a lower cell potential in fuel cell mode and a higher cell potential in electrolysis mode than T-NMPC-Power. This behavior is consistent with the observed trend in electrical work under the two control strategies. Figure 6 also shows that the difference in current density between the two control cases was marginal. The accumulated absolute current density was slightly higher in the economic case (1.777 × 10^6^) than in the T-NMPC-Power case (1.771 × 10^6^).

**Figure 6:**
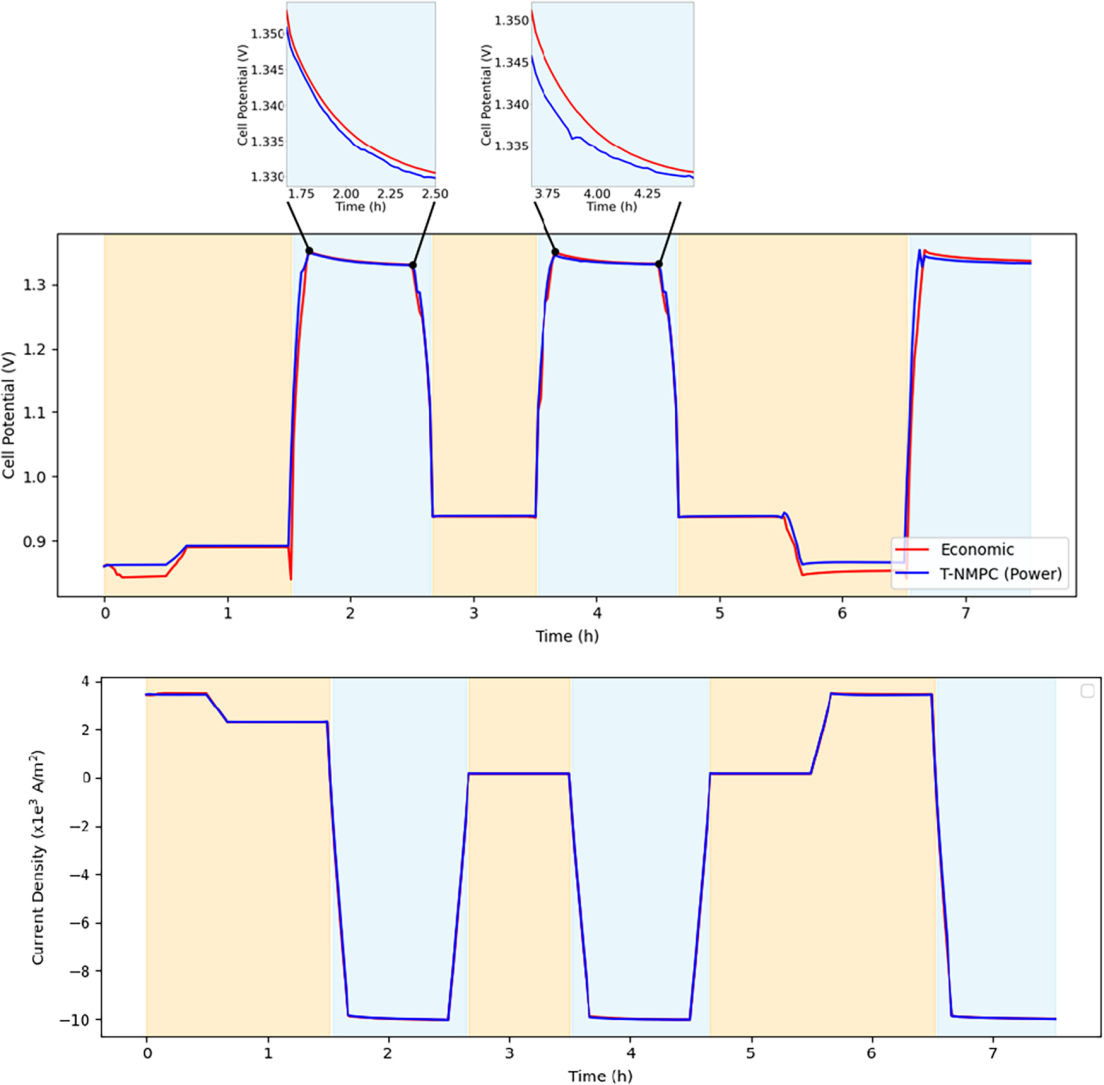
Cell potential and current density when controlled using T-NMPC-Power (blue line) and E-NMPC (red line). The operating modes are distinguished by background shadow color (blue: electrolysis; yellow: fuel cell). The later figures follow the same rule.

Figure 7 illustrates the overall increasing trend of fuel-side reactant conversion because the reaction rate is dependent on the current density. Figure 9 shows the splitter behavior where the feed splitter recycle ratio to the fuel side is lower in the economic control case, while the sweep gas splitter recycle ratio to the oxygen side remains similar. These trends can be attributed to differences in conversion behavior. When operating in fuel cell mode, water is removed from the product stream in a flash vessel, and, to conserve H_2_, the recycle split fraction is higher. On the other hand, in electrolysis mode, H_2_ recycling is avoided to prevent mixing with the makeup stream containing mainly H_2_O, which results in the condenser splitter recycle ratio approaching zero.

**Figure 7:**
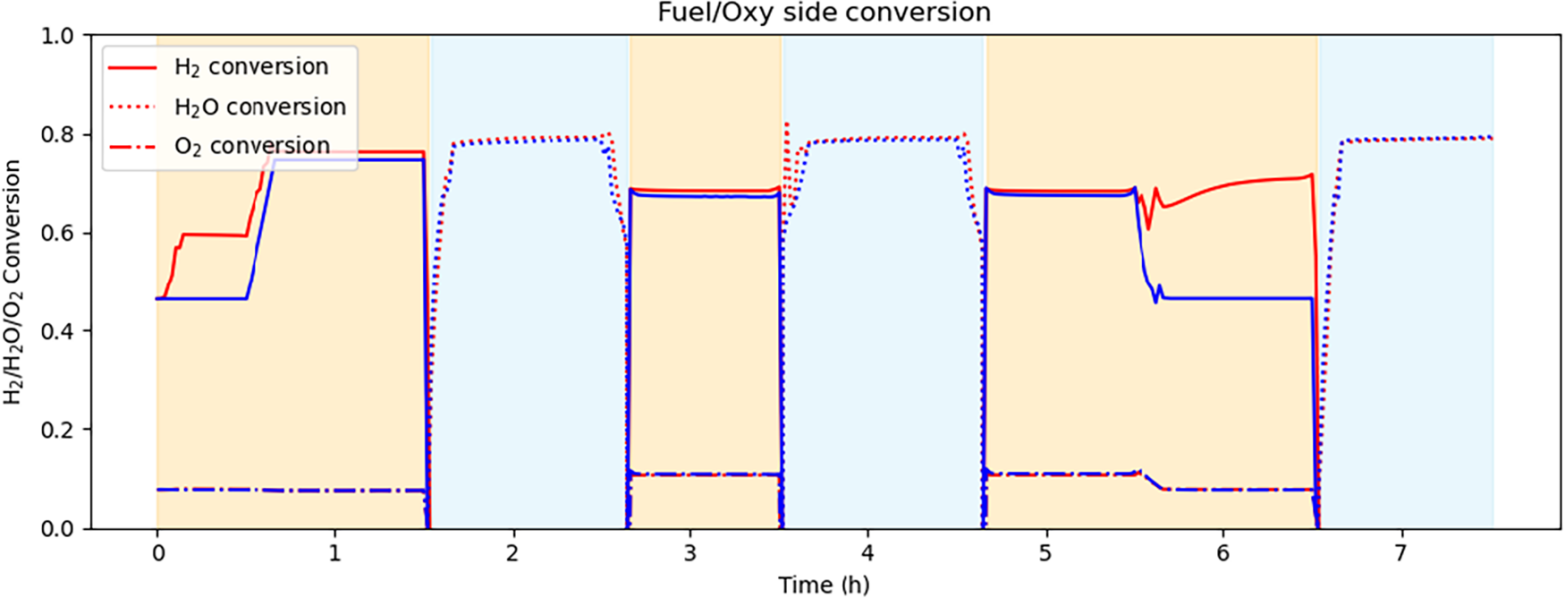
SOC fuel and oxygen-side reactant conversion.

Figure 8 presents the temperature profiles on both the fuel and oxygen sides. Under E-NMPC, the inlet temperature was lower due to the reduced heat input from the trim heaters. However, a more pronounced temperature increase was observed in E-NMPC, particularly on the fuel side, driven by the heat released from the electrochemical reactions and the higher reactant conversion (Figure 9).

**Figure 8:**
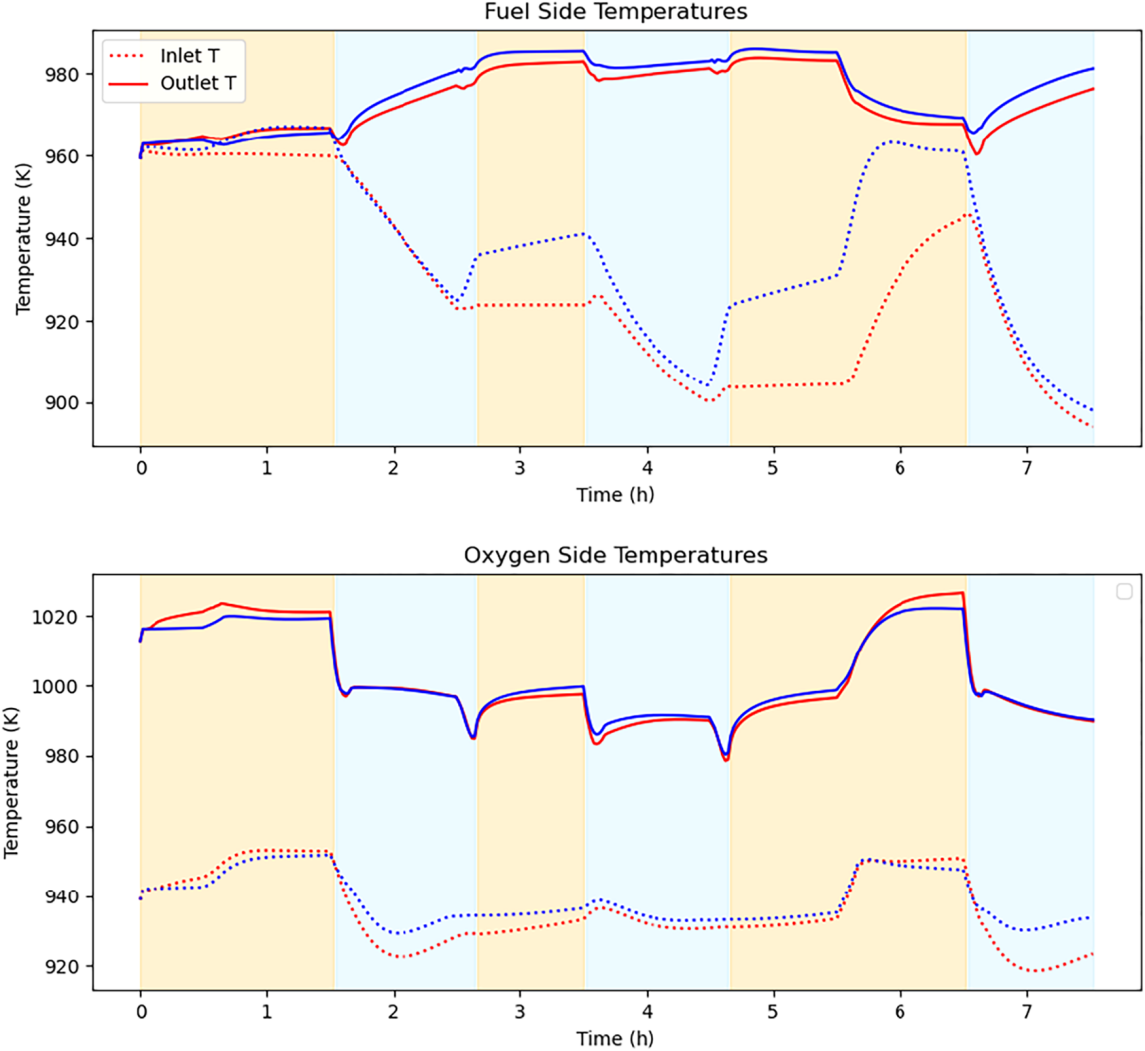
SOC fuel and oxygen-side inlet/outlet temperatures.

**Figure 9:**
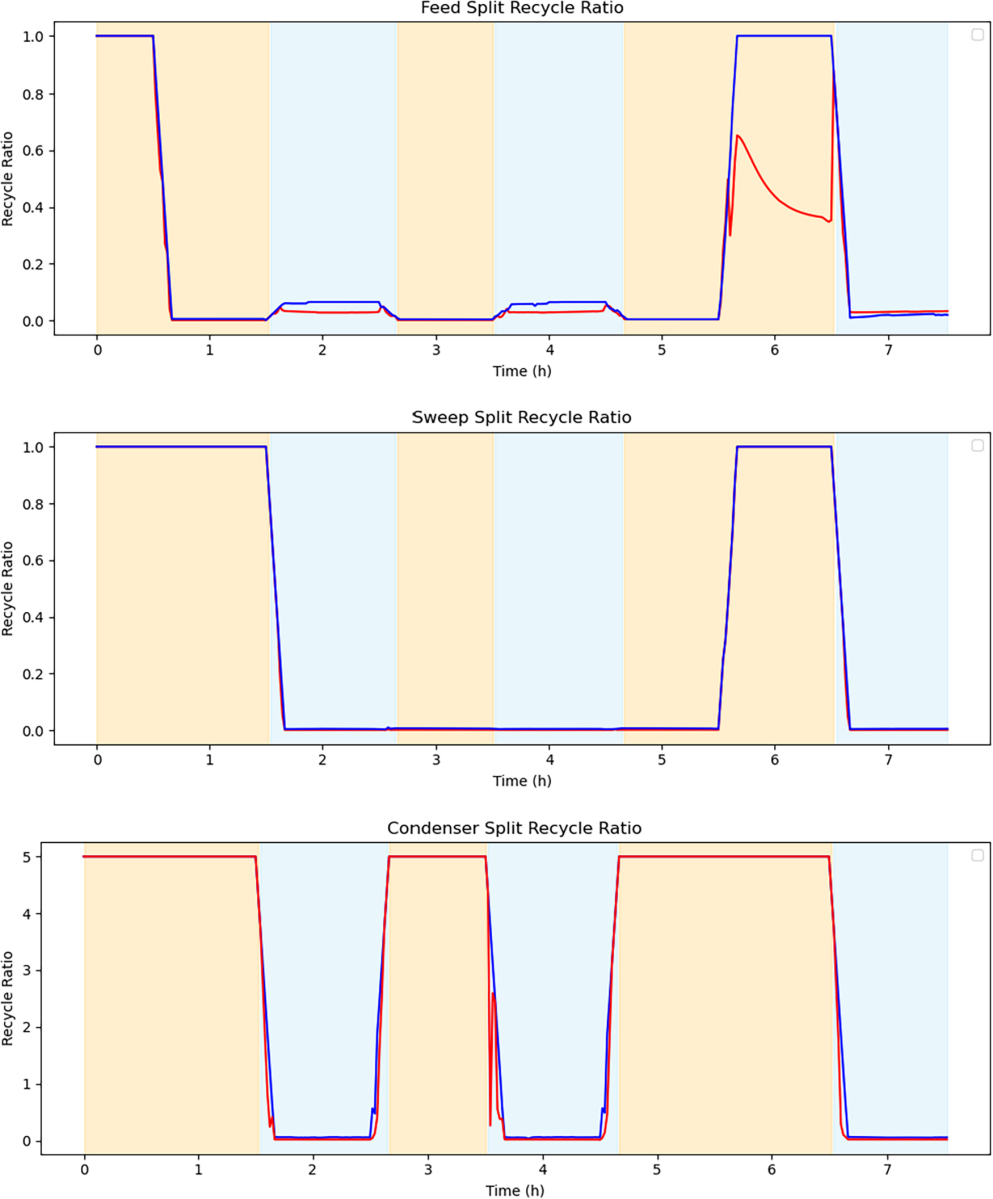
Splitter recycle ratio for the feed, sweep, and condenser splitters.

## 5 Conclusions

In this paper, we proposed an economic NMPC formulation for the optimal control of a reversible solid oxide cell (rSOC) system. E-NMPC was applied to a dynamic model of the rSOC flowsheet using realistic electricity price data based on locational marginal pricing (LMP). The system’s transitions between operational modes posed significant numerical challenges for the control problem, especially due to rapid changes in electricity set-points. To mitigate numerical challenges during mode transitions, we systematically analyzed the discretization strategy and introduced tailored variable transformations that improved model conditioning and solver performance. The modified model allowed us to make operational decisions while making challenging transitions between fuel cell and electrolysis modes.

Based on the work of Li et al.,^17^ a modified tracking NMPC formulation was developed to address the practical need to match electricity market commitments. The E-NMPC approach was compared against the set-point tracking NMPC for the rSOC system. E-NMPC was able to save 4% more hydrogen compared to set-point tracking NMPC while producing the same amount of electricity. Also, E-NMPC yielded a marginal increase in hydrogen production compared to set-point tracking NMPC when the cell was in electrolysis mode. E-NMPC was able to reallocate the total available electrical energy from the grid by decreasing the electrical consumption of the feed and sweep heaters. The rSOC operated using the E-NMPC approach has a higher conversion of the feed compared to set-point tracking NMPC.

The potential of the integration of a battery with the rSOC is also investigated. The analysis considered a battery that can partially handle load from the grid, giving the SOC some degree of freedom to prioritize the economic performance over demand satisfaction. However, in the case of 1 *MW* battery flow limitation, the battery provided marginal improvement in economic performance. A bigger battery was also investigated, which provided greater economic benefit; however, this benefit was the result of running down the charge level of the battery without making provisions to recharge it.

Future work involves detailed battery modeling with terminal constraints on recharging the battery to analyze hybrid systems. Moreover, to reduce the average computational delay of the NMPC CPU-time, our future plans are to incorporate advanced-step NMPC strategies^30,31^ strategies, where the control problem is solved offline and corrected online through sensitivity analysis, thus leading to very short computational delays. In addition, the switching cycles for the rSOC system should also be reformulated as part of regular, periodic behavior that does not converge to a steady state, but to a *cyclic steady state*, which also needs to be incorporated within NMPC and E-NMPC formulations. Such optimization models must also compensate for model mismatch and disturbances. These E-NMPC formulations were derived, analyzed and applied in^8,12,18^ and will be considered in future rSOC case studies. Along with these studies, our E-NMPC formulations can also be extended to utility plants with multiple power generating assets.

## Footnotes

1 This error could be remedied by use of an upwind finite-volume scheme.

2 Hydrogen consumed is not equal to the negative of hydrogen produced. In electrolysis mode, the hydrogen in the out stream is sent for further compression and drying, so it is counted in the hydrogen produced. However, in fuel cell mode, the out stream acts as a purge stream to avoid buildup of inert gases in the system, so any hydrogen in that stream is lost.

## Nomenclature

**Acronyms Description**
rSOC: reversible solid oxide cell
SOFC: solid oxide fuel cell
SOEC: solid oxide electrolyzer cell
TPB: triple phase boundary
NMPC: nonlinear model predictive control (E-Economic;T-Tracking)
LMP: locational marginal pricing
PDE: partial differential equation
DAE: differential algebraic equation
MV: manipulated variable
CV: controlled variable
**Symbol Description**
ρ_mol_: molar density [mol/m^3^]
*P*: gas pressure [Pa]
*R*_u_: universal gas constant [J/mol/K]
*T*: gas temperature [K]
*C*: concentration [mol/m^3^]
*y*: mole fraction
*v*: velocity [m/s]
*w*: state and control variables in stage costs
*L*: length of channel [m]
*J*: molar flux [mol/m^2^/s]
ρ_e_: internal energy density [J/m^3^]
*H*: molar enthalpy [J/mol]
*h*: heat transfer coefficient [W/m^2^/K]
*F*: molar flow rate [mol/s]
*q*: energy flux [W/m^2^]
ρ: resistivity [Ω·m]
*j*: current density [A/m^2^]
*k*: thermal conductivity [W/m/K]
ε: void fraction
*l*: thickness of layer [m]
*r*: reaction rate [mol/m^2^/s]
ν: stoichiometric coefficient
η_act_: activation potential at TPB [V]
Δ*S*: entropy of reaction [J/K]
*N*_cell_: number of cells
**Subscript Description**
*i*: species i
*x*, *y*, *z*: direction *x*, *y*, *z*
*x*_0_: at *x*_0_ boundary
*x*_1_: at *x*_1_ boundary
g: gas
s: solid
A: side A
B: side B
ftpb: fuel triple phase boundary
otpb: oxygen triple phase boundary
rxn: reaction
*k*: fuel or oxygen TPB
